# The Unknown Oldowan: ~1.7-Million-Year-Old Standardized Obsidian Small Tools from Garba IV, Melka Kunture, Ethiopia

**DOI:** 10.1371/journal.pone.0145101

**Published:** 2015-12-21

**Authors:** Rosalia Gallotti, Margherita Mussi

**Affiliations:** 1 Dipartimento di Scienze dell'Antichità, Università di Roma "La Sapienza", Via dei Volsci 122, 00185 Rome, Italy; 2 Italian Archaeological Mission at Melka Kunture and Balchit, Rome, Italy; 3 Université Bordeaux 1 –UMR5199 PACEA-PPP, Bâtiment B18 allée Geoffroy Saint-Hilaire CS 50023 F—33615 PESSAC CEDEX, France; Universidade do Algarve, PORTUGAL

## Abstract

The Oldowan Industrial Complex has long been thought to have been static, with limited internal variability, embracing techno-complexes essentially focused on small-to-medium flake production. The flakes were rarely modified by retouch to produce small tools, which do not show any standardized pattern. Usually, the manufacture of small standardized tools has been interpreted as a more complex behavior emerging with the Acheulean technology. Here we report on the ~1.7 Ma Oldowan assemblages from Garba IVE-F at Melka Kunture in the Ethiopian highland. This industry is structured by technical criteria shared by the other East African Oldowan assemblages. However, there is also evidence of a specific technical process never recorded before, i.e. the systematic production of standardized small pointed tools strictly linked to the obsidian exploitation. Standardization and raw material selection in the manufacture of small tools disappear at Melka Kunture during the Lower Pleistocene Acheulean. This proves that 1) the emergence of a certain degree of standardization in tool-kits does not reflect in itself a major step in cultural evolution; and that 2) the Oldowan knappers, when driven by functional needs and supported by a highly suitable raw material, were occasionally able to develop specific technical solutions. The small tool production at ~1.7 Ma, at a time when the Acheulean was already emerging elsewhere in East Africa, adds to the growing amount of evidence of Oldowan techno-economic variability and flexibility, further challenging the view that early stone knapping was static over hundreds of thousands of years.

## Introduction

The Oldowan has long been thought to have been static, with limited internal variability, embracing techno-complexes characterized by percussion materials, cobble tools and unmodified flakes [1–4].

Since the 1990s, new discoveries and new research based on technological analysis of lithic collections brought this view into question [5–15]. They suggested that Oldowan industries displayed a greater technological skill and internal variability than had been surmised. The new studies found that Oldowan artifacts display 1) good knowledge of the physical mechanisms involved in conchoidal fractures; 2) controlled percussive motion; 3) an understanding of how the suitability of raw materials for knapping varies; 4) adaptation to matrix geometry for different purposes; and 5) a variety of flaking methods. They also argued that much of the observed inter-site variability was due to the quality, knapping suitability, size and shape of the raw materials used [9,16–19].

Notwithstanding this re-evaluation of the early knappers’ skills, the manufacture of small tools–flakes with one or more edges modified by retouch–is only an occasional component of the Oldowan techno-complexes. When present at all, small tools are found in very small percentages and do not show any standardization. In late Pliocene sites, retouched items at EG10 and EG12 at Gona (in the Afar region) account respectively for just 2.5% and 4% of the whole flakes, and 2.5% at Lokalalei 2C in West Turkana [3, 4, 10]. They are totally absent in the assemblages retrieved at A.L. 894 in Hadar, at Lokalalei 1 in West Turkana, at Fejej FJ-1a and at the Omo sites [7, 8, 10, 20].

At Olduvai itself, a recent technological review of the Oldowan industries [9] proves that many small tools previously identified by Leakey [1] and Kimura [21] are actually flakes with pseudo-retouching due to post-depositional processes. Accordingly, they can no longer be considered as human-modified by retouch. Although more frequent than at late Pliocene sites, around 1.8–1.7 Ma retouched flakes are a minor component of Bed I assemblages, e.g. at DK, FLK Zinj and FLK North Levels 1–2 (8–12% of the whole flakes) [9]. Besides, they generally display irregular and variable morphologies without any standardization [9, 19].

**Fig 1:**
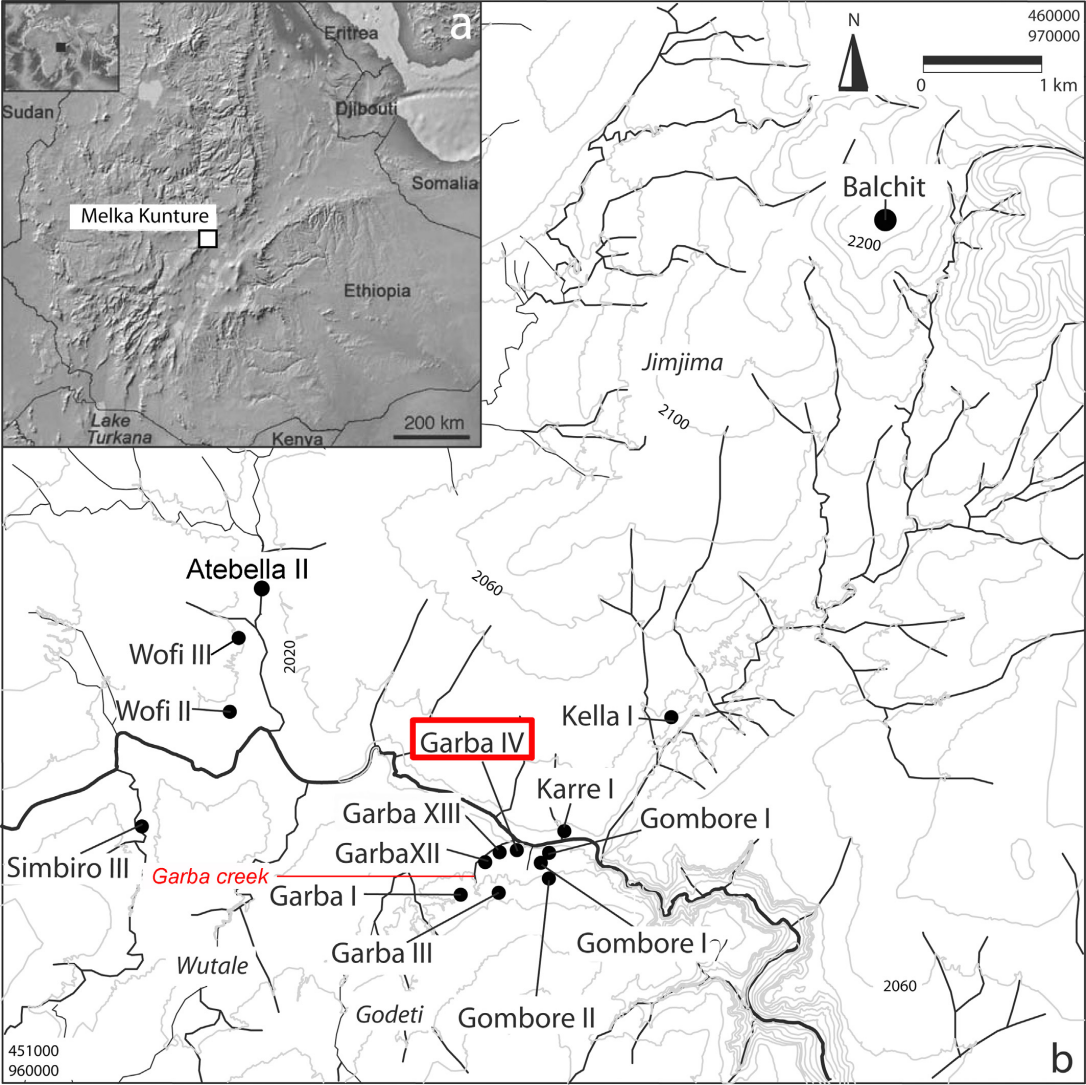
a: Map of Ethiopia and neighboring countries, showing the location of Melka Kunture on the shoulder of the Main Ethiopian Rift (modified after USGS National Map Viewer); b: Map of the Melka Kunture area, showing the location of the archaeological sites (vector restitution of the 1:50,000 topographic map by R. Gallotti).

Summing up, intentionally retouched flake edges are known to occur only occasionally in the Oldowan, or to be altogether absent. This view is now challenged by the findings of our recent research at Garba IVE-F at Melka Kunture, in the Upper Awash Valley in the Ethiopian highlands (Fig 1A). We present here the earliest known evidence of systematic production of standardized obsidian small tools in the Oldowan.

## Materials and Methods

The lithic collections from Garba IVE-F were analyzed in their entirety (1415 specimens corresponding to artifacts and unworked material assemblages). Fieldwork permits and access to the collections are given yearly to Margherita Mussi, director of the Italian Archaeological Mission at Melka Kunture and Balchit, by the Authority for Research & Conservation of Cultural Heritage of the Ethiopian Ministry of Culture & Tourism. These collections, studied in 2014, are permanently stored and accessible to study at the National Museum of Ethiopia in Addis Ababa under regulations established by the Authority for Research and Conservation of the Cultural Heritage.

The reference to the research permit for 2014 fieldwork and collection analysis is: 08/709-15/001 delivered on September 12, 2014, by Desalegn Abebaw, Director, Cultural Heritage & Research Directorate, Authority for Research and Conservation of the Cultural Heritage, Addis Ababa, Ethiopia.

### Geo-Chronological and Archaeological Context

Melka Kunture is located in the Upper Awash Valley, 50 km south of Addis Ababa on the western border of the Main Ethiopian Rift, in a semi-graben depression of the Ethiopian Plateau, between 2000 and 2200 m asl (Fig 1A). The Awash basin is drained by the Upper Awash River and its tributaries, and is bounded by Pliocene volcanoes: the Wachacha and Furi to the North, the Boti and Agoiabi to the South. The Pleistocene reactivation of border faults led to several episodes of subsidence in the semi-graben. This in turn increased the sedimentation rate, while during eruptions pyroclastic material was added to the load transported by the river system [22]. This is better documented by the alluviums of the right bank tributaries. On the left bank, there is a record of dismantled superficial formations between the Awash and the volcanic centers.

Volcanism was characterized by multiple and often violent eruptions related to the Late Cenozoic evolution of the Ethiopian Rift. The major volcanic events started 5 to 4 Ma ago, but later eruptions also modified the environment when hominin groups were already present. The Awash river was able to re-establish its course after each volcanic episode. The water flow of the main river and of its tributaries reworked and transported loads of sediments, including volcanic material, that buried and preserved archaeological sites.

The piling of alluviums, volcano-derived sediments and direct tephric inputs built up the Melka Kunture Formation [23]. Recent dating documents a human occupation of this part of the Upper Awash Valley between the end of Olduvai Polarity Subzone and at least the Brunhes Matuyama Reversal [24].

Most of the archaeological sites were discovered in the core area of the semi-graben, clustering over some 100 km^2^. The Palaeolithic sequence starts with the Oldowan at Karre I, Gombore I, Garba IVE-G, and Gombore Iγ; it continues with the early Acheulean at Garba IVD; with the Middle Acheulean at Gombore II, Garba XII, Garba XIII, Atebella II, and Simbiro III; and with the Late Acheulean at Garba I and Garba IIIC. Garba IIIB is the most important Middle Stone Age site. The Late Stone Age is found eroding from superficial deposits at Wofi II, Wofi III, and Kella I (Fig 1B) [25–29].

### The Garba IV site

**Fig 2:**
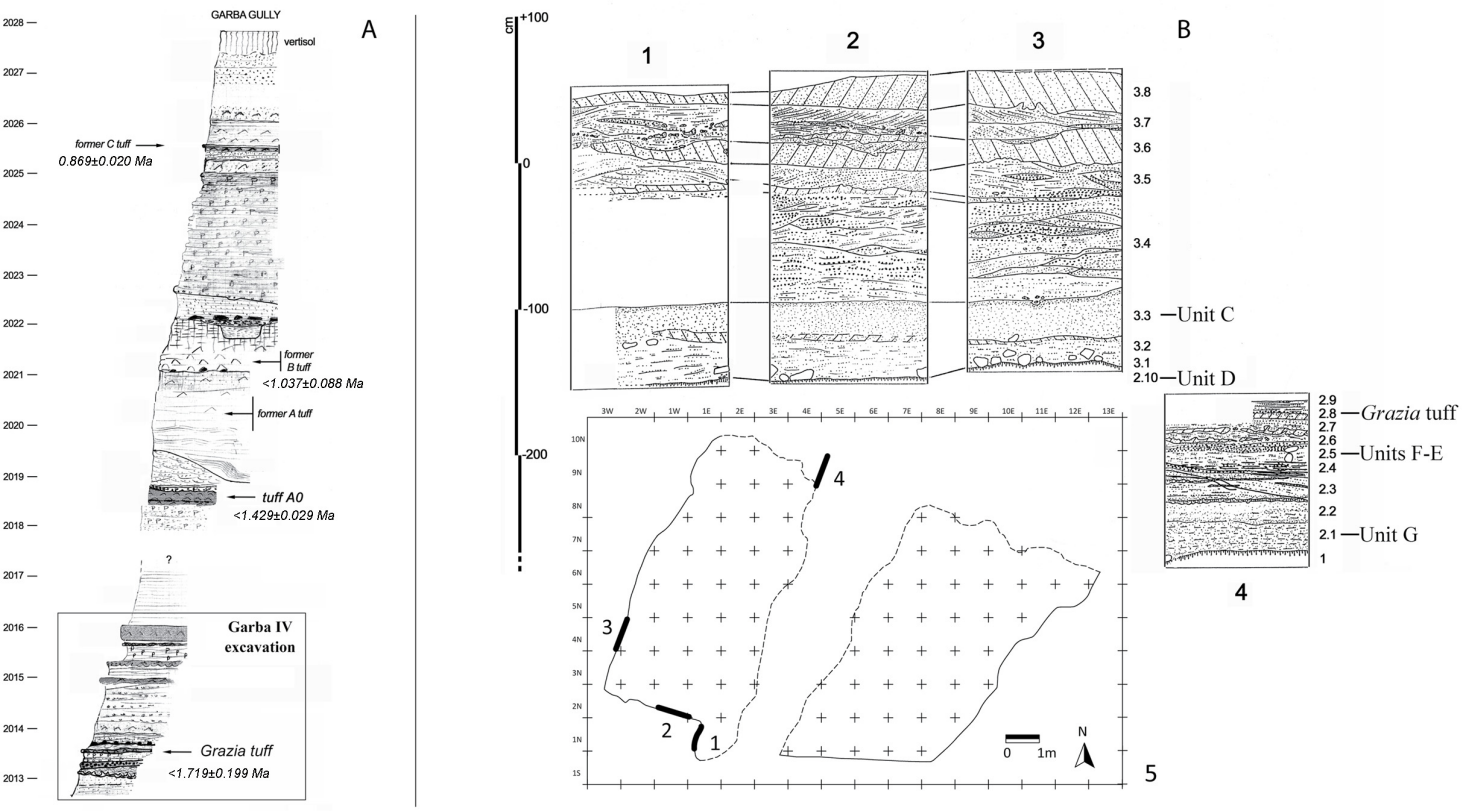
A: The Melka Kunture Formation along the Garba gully (after 23, revised; dates after 24); B: 1–5: Stratigraphic sections at Garba IV (after 23, revised). Stratigraphic Unit 1, at the bottom of the sequence, is a layer of greenish silty sands and a typical sediment gravity flow deposit.

**Fig 3:**
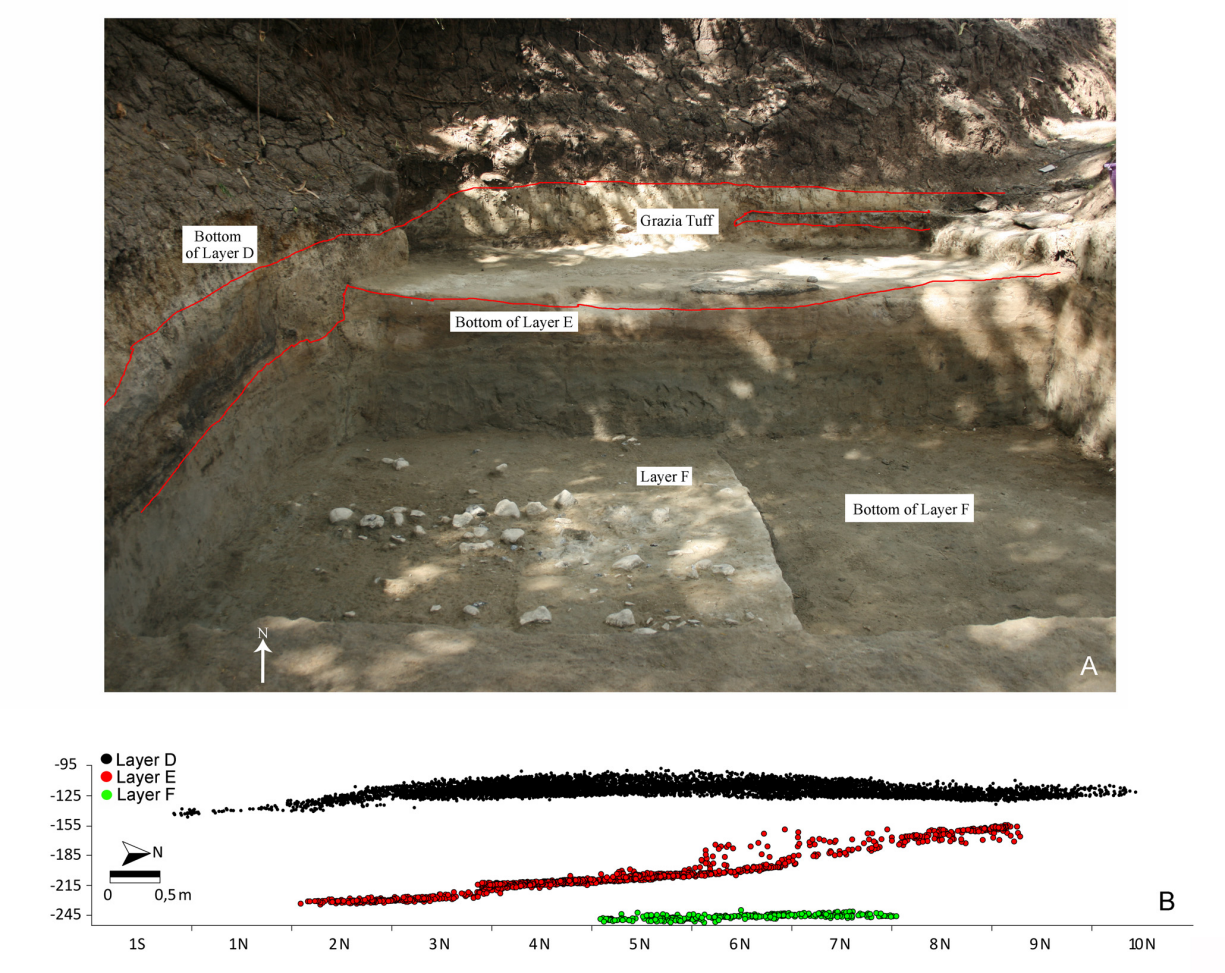
A: Garba IV during 2009 excavation; B: S-N projections of lithic artifacts and faunal remains. Photos and map by R. Gallotti.

Garba IV, on the right bank of the Awash at the confluence of the Garba creek (Fig 1B), was discovered in 1972 by Jean Chavaillon, who excavated it from 1973 to 1982 [30–31]. It is a key site for understanding the Oldowan and the shift from the Oldowan to the Acheulean on the Ethiopian plateau. The deposit belongs to the lowest parts of the Melka Kunture Formation which dates from the Lower Pleistocene. In an approximately 3-meter stratigraphy, three stratigraphic units were recognized in sedimentary fluvial series, and several archaeological horizons were discovered [23]. The sequence lies below tuff A0, dated to <1.429 ± 0.029 Ma [24], which accordingly also caps layers C and D. The lithic assemblage of layer D documents the emergence of the Acheulean at Melka Kunture at approximately 1.5 Ma [27]. The Grazia tuff sandwiched between layer D and the underlying layer E is dated to <1.719 ± 0.199 [24] (Figs 2 and 3). Layers E and F, located below the Grazia Tuff, are included by Tamrat et al. [32] in the normal polarity interval (N1), which has been interpreted as the end of the Olduvai subchron.

Stratigraphic Unit 2 is divided into 10 subunits. From bottom to top:

2-1 Silty sands of a sediment gravity flow deposit with the lowermost archaeostratigraphic unit, i.e. unit G.
2-2 Light-gray ashy sands indicative of a sediment-gravity to plane-bed flow deposit.
2-3 Silty sands of a plane-bed flow deposit.
2-4 Sands, mixed lenses from coarse to fine.
2-5 Grey pumiceous silty sands including archaeostratigraphic units F and E.
2-6 Gravels with obsidian granules.
2-7 Pumiceous sands with coarsely stratified pumice, probably derived from a distant airfall ash.
2-8 White tuff of a distal direct airfall ash (Grazia tuff).
2-9 Fine sandy layer.
2-10 Green silty sands of a sediment gravity flow deposit.

Stratigraphic Unit 3 is composed of eight subunits. From bottom to top:

3-1 Clast-supported massive gravel deposit that constitutes archaeostratigraphic unit D.
3-2 Upward refining (from coarse to fine) bedded sands.
3-3 Coarse massive sands containing archaeostratigraphic unit C.
3-4 Coarse sands and gravels with fine interbedded stratification, cradles and lenses, suggesting the lateral evolution of ephemeral shallow channels.
3-5 Cineritic layer of irregular thickness.
3-6 Redeposited white tuff, muddy flow coulee type with surf structures.
3-7 Obliquely stratified sands indicative of low flow regime.
3-8 White sandy tuff.

Subunits 5 to 8 form a single reworked tuff unit.

**Fig 4:**
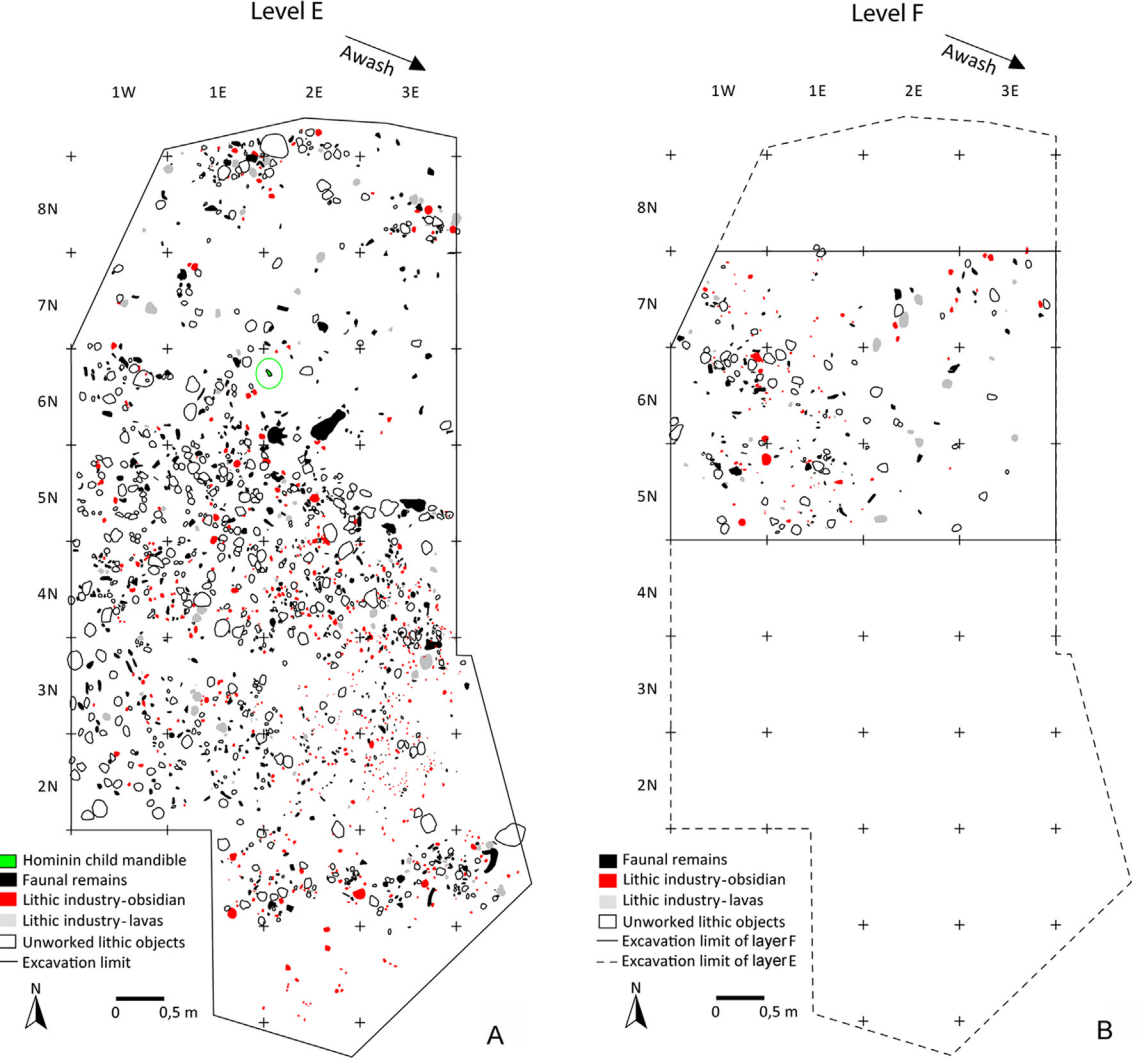
Garba IV. Horizontal maps of layers E (A) and F (B). Maps by R. Gallotti.

In 1982, layer E was tested over 4m^2^ [33, 34]. In this layer, the fragmented mandible of a two- or three-year-old *Homo erectus s*.*l*. child was discovered [35–37] together with lithics and faunal remains. In 2005, 2008 and 2009, new excavations explored both layer E and the underlying layer F over approximately 34 m^2^ and 12 m^2^, respectively. Every single item was recovered, including unworked lithic items. Layer E yielded 504 unworked lithic objects, 718 artifacts and 774 faunal remains; layer F yielded 80 unworked lithic objects, 113 artifacts and 110 faunal remains. The spatial data of each object ≥1 cm were recorded in three dimensions (Fig 4). Hundreds of small flakes and indeterminable fragments less than 1cm long were also collected by systematically sifting the sediment of each layer by half square-meters sections.

### The technological analysis

A qualitative rather than a quantitative approach is used to assess the range of variation and to characterize the modes of lithic productions. Following the chaine operatoire approach, the term lithic production is used here to describe a sequence of varied technical actions and reductive phases resulting in a techno-economic process; that is to say, this approach includes all the technical sequences performed and all the related technical and cognitive skills involved in tool production [38–44].

A classification of the cores, which is particularly informative when assessing knapping methods and techniques, was performed by identifying the number of flaking surfaces, the direction of flaking, the presence or absence of a distinct prepared striking platform, and the angle between the striking platform and the flaking surface. Taking these attributes into account, the core analysis facilitated the identification of exploitation modalities, making it possible to understand the management of material volume and the presence or absence of a hierarchical organization of the surfaces. The flake analysis takes into account the type of butt, the number and direction of negative scars on the dorsal face, the shape and cross-section, the correspondence between morphological and debitage axis, the presence of overshot/hinged removals, the presence of retouch and its location and type, and the presence of a correspondence among shape, size and debitage method.

Each analyzed lithic object (both knapped and unworked ones) was lithologically characterized to establish the overall composition of raw materials, as well as the provisioning and exploitation patterns as potential sources of archaeological assemblage variability. Additionally, unworked objects were classified in terms of their natural shape and size in order to determine the range of available shapes and dimensions. Convexity or angularity of the surfaces could have played a role in the choice of a specific production activity or debitage method.

## Results: Technology of the Garba IVE-F Stone Assemblages

**Fig 5:**
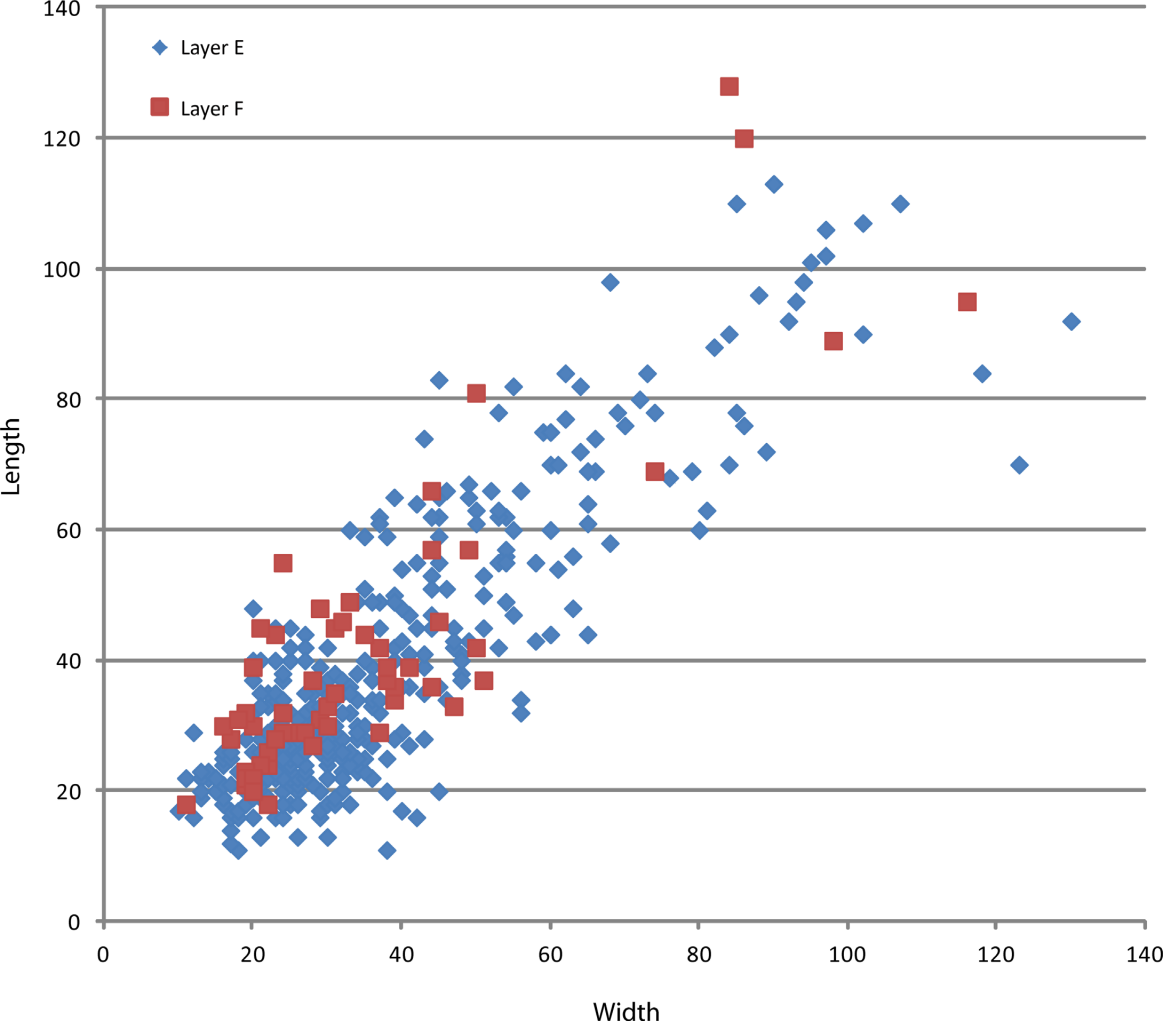
Size distribution (mm) of the cores, whole flakes and retouched flakes in layers E and F.

The assemblages from layers E and F (Tables 1 and 2) have been deposited within a relatively short period of time [23]. Both are produced mainly on obsidian cobbles and pebbles. All stages of reduction and manufacture are found. The comparative analysis of the two series showed very similar technical patterns: 1) the presence of a single chaine operatoire, i.e. small debitage; 2) the same selection patterns in raw material exploitation; 3) a single provisioning system; 4) small debitage methods governed by the same technical criteria; 5) in both instances flake technical patterns in accordance with debitage methods; 6) in both series the same focus in flake retouching; 7) metrical similarities in size distributions of cores, whole flakes, and retouched flakes (Table 3; Fig 5). Accordingly, we will analyze them jointly.

**Table 1:** Garba IVE. Components of the lithic assemblage. OBS: obsidian.

Component	OBS	LAVAS				Total
		*Aphyric lavas*	*Porphyritic lavas*	*Microdoleritic basalt*	*Welded ignimbrite N*.*1*	
Cobble	3	114	3	5	104	**229**
Broken cobble	-	14	1	-	9	**24**
Pebble	10	26	-	1	21	**58**
Natural element	-	33	1	-	21	**55**
Natural fragment	2	56	-	1	67	**126**
Small block	-	2	1	-	2	**5**
Block	-	4	-	-	3	**7**
**Total unworked material**	**15**	**249**	**6**	**7**	**227**	**504**
Core	46	34	1	-	-	**81**
Core fragment	6	-	-	-	-	**6**
Whole flake	194	61	-	-	-	**255**
Broken flake	116	18	-	-	-	**134**
Retouched flake	66	1	-	-	-	**67**
Indeterminable fragment	140	35	-	-	-	**175**
**Total artifacts**	**568**	**149**	**1**	**-**	**-**	**718**
**TOTAL**	**583**	**398**	**7**	**7**	**227**	**1222**

**Table 2:** Garba IVF. Components of the lithic assemblage. OBS: obsidian.

Component	OBS	LAVAS				Total
		*Aphyric lavas*	*Porphyritic lavas*	*Microdoleritic basalt*	*Welded ignimbrite N*.*1*	
Cobble	3	21	-	4	4	32
Broken cobble	-	7	-	-	2	9
Pebble	4	8	-	2	2	16
Natural element	-	8	-	2	6	16
Natural fragment	1	-	-	-	6	7
Small block	-	-	-	-	-	-
Block	-	-	-	-	-	-
**Total unworked material**	**8**	**44**	**-**	**8**	**20**	**80**
Core	6	5	-	-	-	11
Core fragment	2	1	-	-	-	3
Flake	39	1	-	-	-	40
Broken flake	22	2	-	-	-	24
Retouched flake	7	-	-	-	-	7
Indeterminable fragment	22	5	1	-	-	28
**Total artifacts**	**98**	**14**	**1**	**-**	**-**	**113**
**TOTAL**	**106**	**58**	**1**	**8**	**20**	**193**

**Table 3:** Dimensions (mm) of cores, whole flakes, undifferentiated retouched flakes, and small pointed tools in layers E and F.

Component		Layer E						Layer F					
		Obsidian			Lavas			Obsidian			Lavas		
		Length	Width	Thickness	Length	Width	Thickness	Length	Width	Thickness	Length	Width	Thickness
**Cores**	Min.	25	22	13	48	37	30	33	47	40	81	44	36
	Max.	102	130	91	110	123	106	128	98	69	95	116	86
	Mean	62.6	57.6	41	77.5	77.6	57.9	65.4	57.3	42.6	88.2	74	60
	Std. dev.	18.5	21.2	16.8	20.5	24.8	22.7	33.3	26.4	12.8	26.4	33.6	25.7
**Flakes**	Min.	11	10	3	18	17	6	18	11	4	-	-	-
	Max.	60	61	29	98	74	40	57	51	34	-	-	-
	Mean	27.7	27	10.1	42.9	40.9	17.6	33.8	28	11.2	23	19	8
	Std. dev.	9.3	9	4.1	18.5	14.3	8.8	9.6	9.9	6.3	-	-	-
**Und. ret. flakes**	Min.	22	21	8	-	-	-	-	-	-	-	-	-
	Max.	74	58	36	-	-	-	-	-	-	-	-	-
	Mean	43.6	37.6	18.7	31	22	10	32	22	12	-	-	-
	Std. dev.	12.8	11.4	6	-	-	-	-	-	-	-	-	-
**Small pointed tools**	Min.	18	13	6	-	-	-	20	20	7	-	-	-
	Max.	30	41	19	-	-	-	29	28	11	-	-	-
	Mean	25.3	25.6	10.7	-	-	-	26.2	24.7	9.2	-	-	-
	Std. dev.	3.5	6.4	3	-	-	-	4.2	3.6	2	-	-	-

### Raw material availability, procurement and selection

Obsidian is the most frequently exploited lithic resource in both layers. It represents 79.4% of the artifacts in layer E and 86.7% in layer F (Tables 1 and 2). The non-obsidian part of the artifact assemblages is on aphyric lava cobbles, which at the time were easily available in the old alluviums of nearby streams [45]. The welded ignimbrite N.1 is also well represented in the alluvium system and accounts for 45% of the unworked sample in layer E and for 20.8% in layer F. This porous, non-homogeneous and non-compact rock, which is unsuitable for knapping, has never been used by knappers in the Melka Kunture sequence. Porphyritic and microdoleritic lavas occur in very low proportion in the Garba IVE-F assemblages (Tables 1 and 2).

Obsidian occurs in low numbers within the unworked material, and is primarily composed of pebbles and small-medium cobbles, which are on the opposite obsidian natural forms abundantly available in the Quaternary alluviums [45]. Accordingly, intense exploitation depleted our unworked assemblage of its obsidian component. The composition of the unworked assemblage, even if impoverished by hominin exploitation, reflects the lithic resource availability in the paleo(channels).

Medium-sized obsidian cobbles and pebbles are present in old alluvial deposits of the Awash and its tributaries [45]. They were produced by the dismantling of the obsidian outcrops located at Balchit, ~7km north-east of Garba IV and at a slightly higher elevation (Fig 1B). This source, dated to 4.37 ± 0.07 Ma [46], belongs to the group of Pliocene rift margin siliceous centers in the Wachacha Formation, located on the western edge of the Main Ethiopian Rift in the Addis Ababa rift embayment. The flat obsidian dome flow crops out over an area of approximately 4 km^2^. This natural glass is easy to break, being compact and homogeneous, hard and poorly porous, and vitreous or finely textured [47–48]. The same elemental composition was recorded in Oldowan and Acheulean obsidian artifacts [49] and in obsidians from alluvial deposits [46–47].

### Debitage

**Fig 6:**
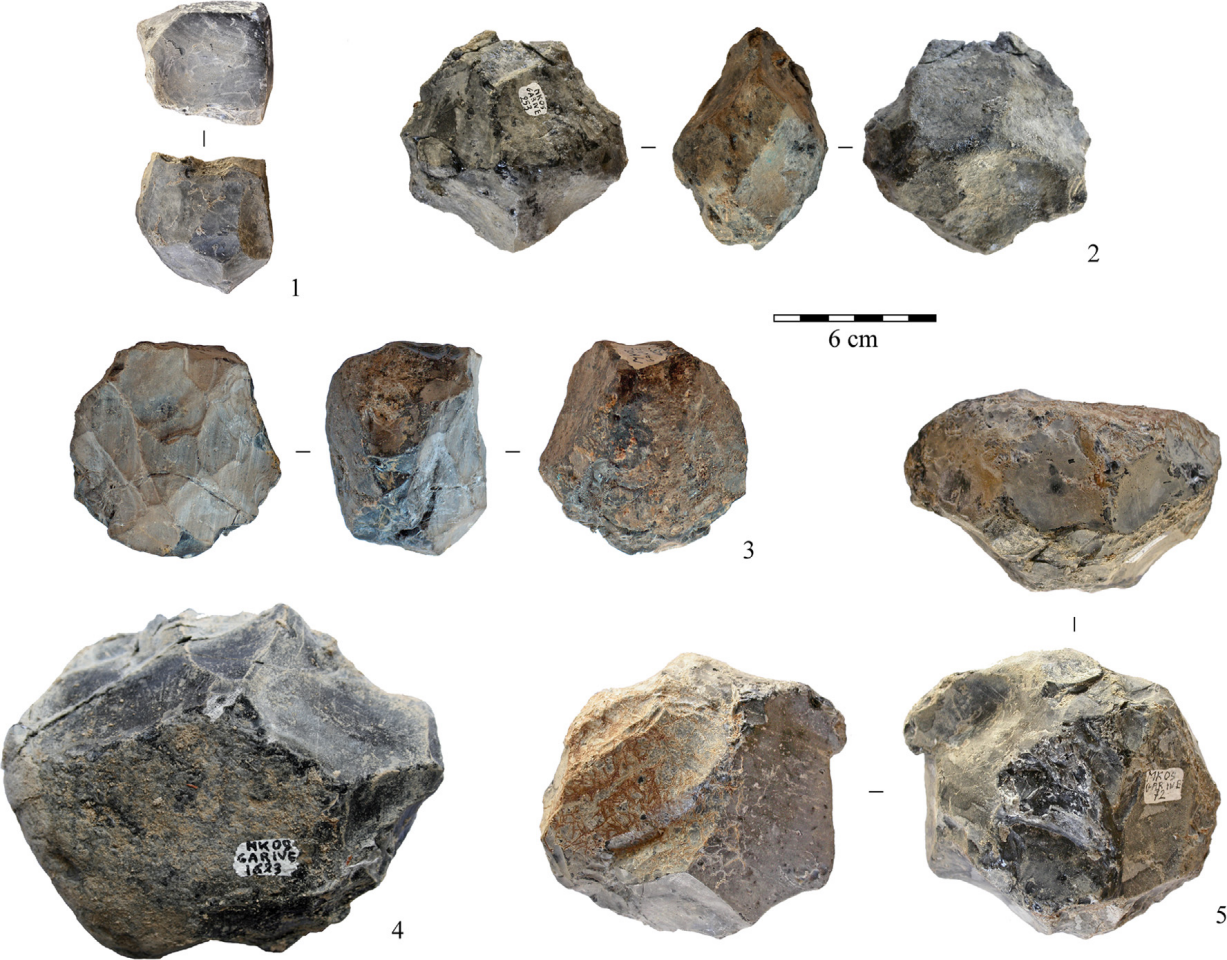
Photographs of selected obsidian cores from Garba IVE-F. 1, 4: unifacial unidirectional cores; 2, 3: centripetal/tangential cores; 5: multifacial multidirectional core. Photos by R. Gallotti.

**Fig 7:**
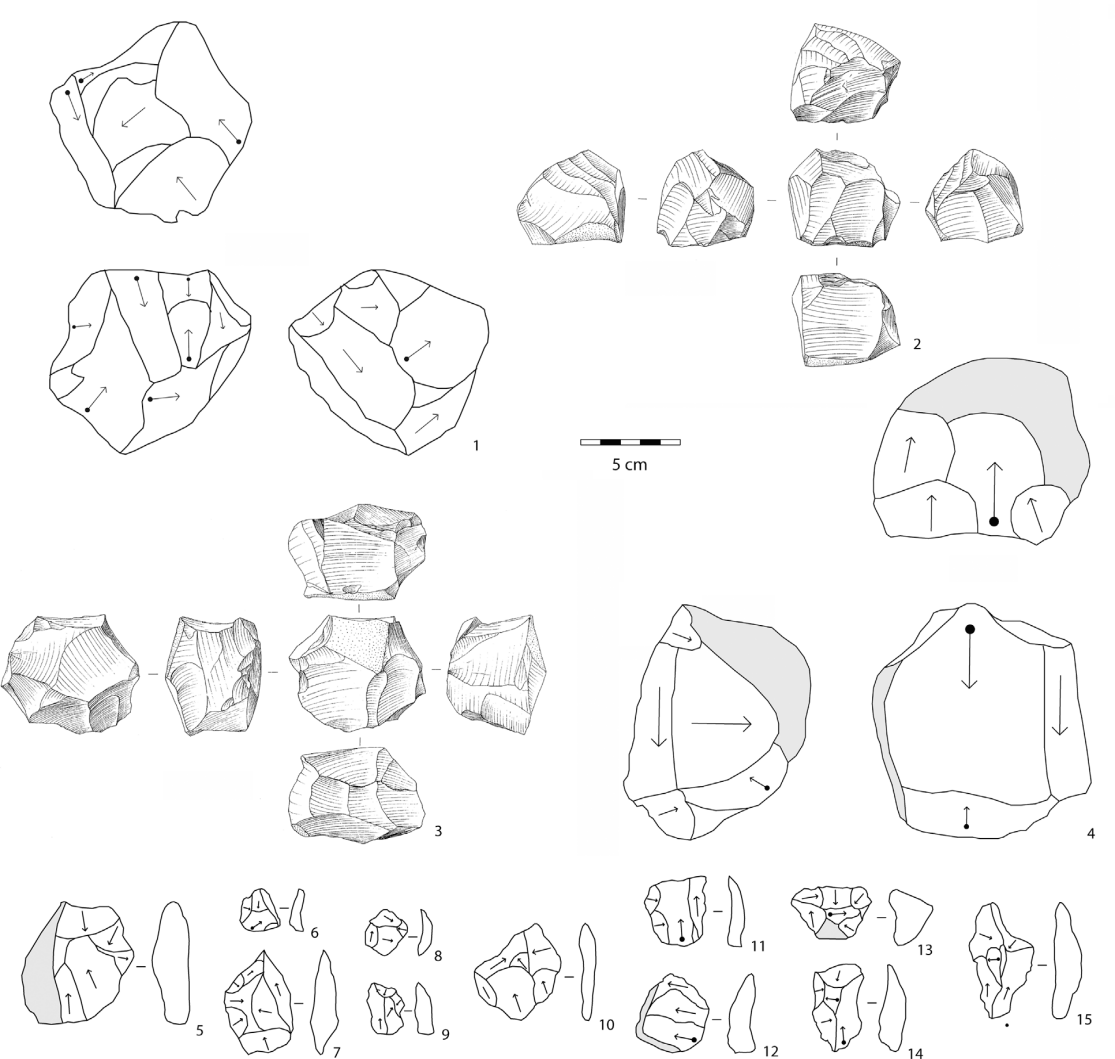
1: multifacial multidirectional irregular core (OBS); 2, 4: multifacial multidirectional cores with major unidirectional flaking surface(s) (2: OBS; 4: ASB); 3: multifacial multidirectional orthogonal core (OBS); 5–9: flakes with multidirectional irregular negative scars on the dorsal face (5: ASB; 6–9: OBS); 10–12: flakes with orthogonal negative scars on the dorsal face (OBS); 13–15: core edge flakes with multidirectional removals (OBS). 2, 3: drawings by M. Pennacchioni. 1, 4–12: technological schemes by R. Gallotti. ASB: aphyric to subaphyric basalt, OBS: obsidian. 2, 3: these drawings have been modified after 34. They have been rearranged and integrated with new ones in this Figure, which is for illustrative purposes only.

**Fig 8:**
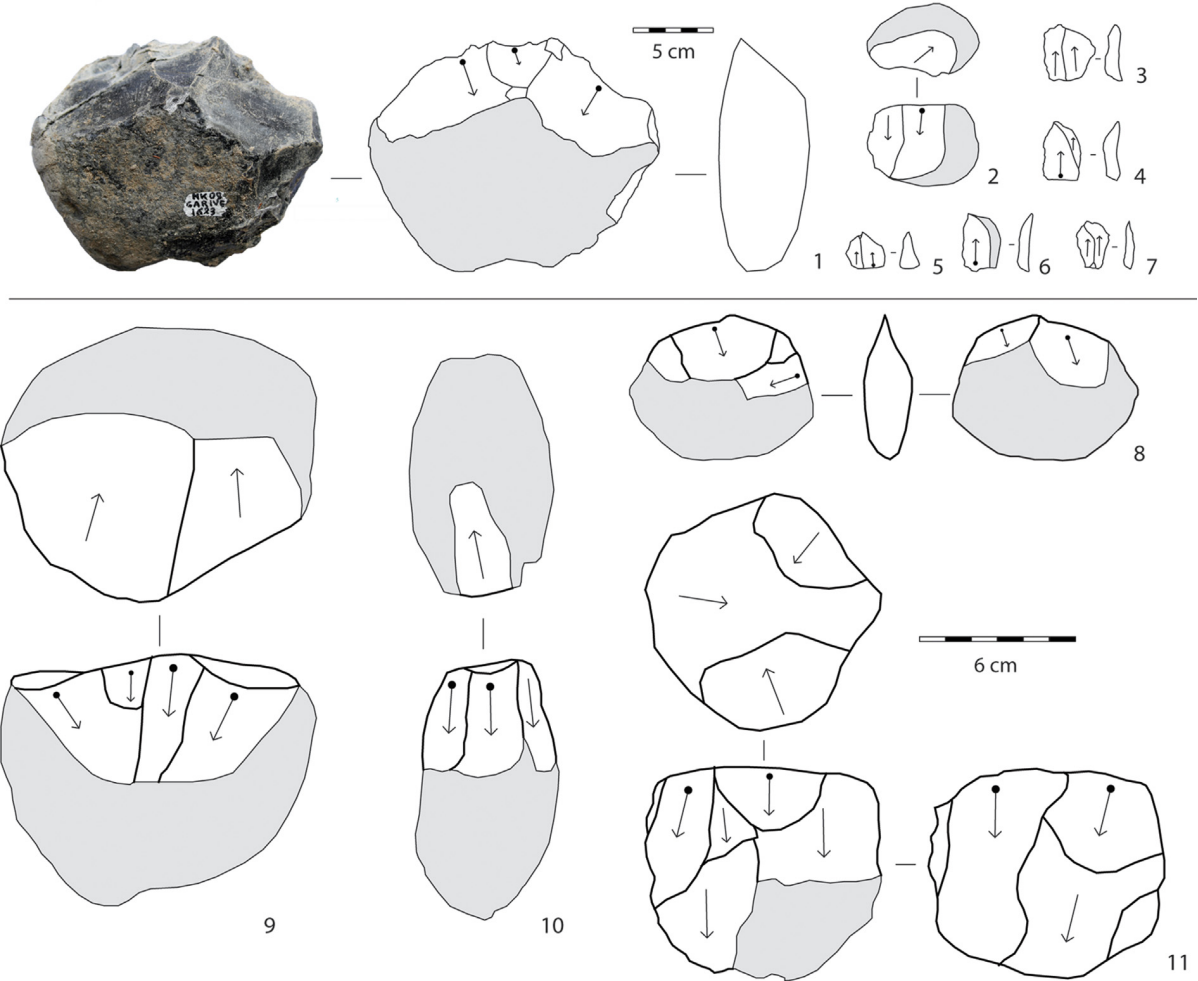
1, 2, 9, 10: unifacial unidirectional cores (1–2: OBS; 9–10: MFL); 3–7: flakes with unidirectional negative scars on the dorsal face (OBS); 8: bifacial partial core (MFL); 11: peripheral unidirectional core (OBS). Photos and technological schemes by R. Gallotti. MFL: Melka Fault lava, OBS: obsidian.

**Fig 9:**
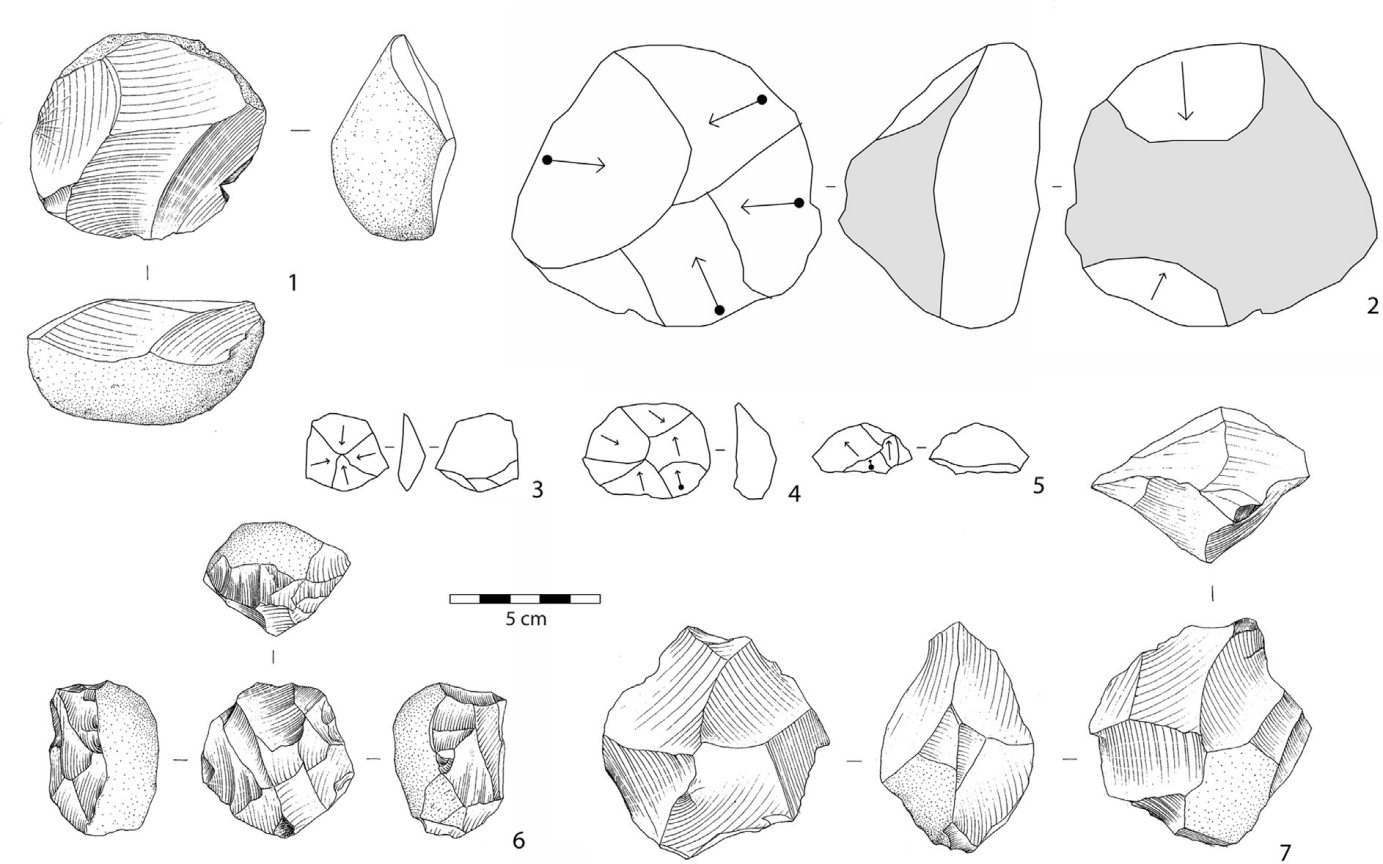
1, 2, 6, 7: centripetal/tangential cores; 3–5: flakes with centripetal/tangential negative scars on the dorsal face. Obsidian. 1, 6, 7: drawings by M. Pennacchioni; 2–5: technological schemes by R. Gallotti. 6, 7: these drawings have been modified after 34. They have been rearranged and integrated with new ones in this Figure, which is for illustrative purposes only.

**Fig 10:**
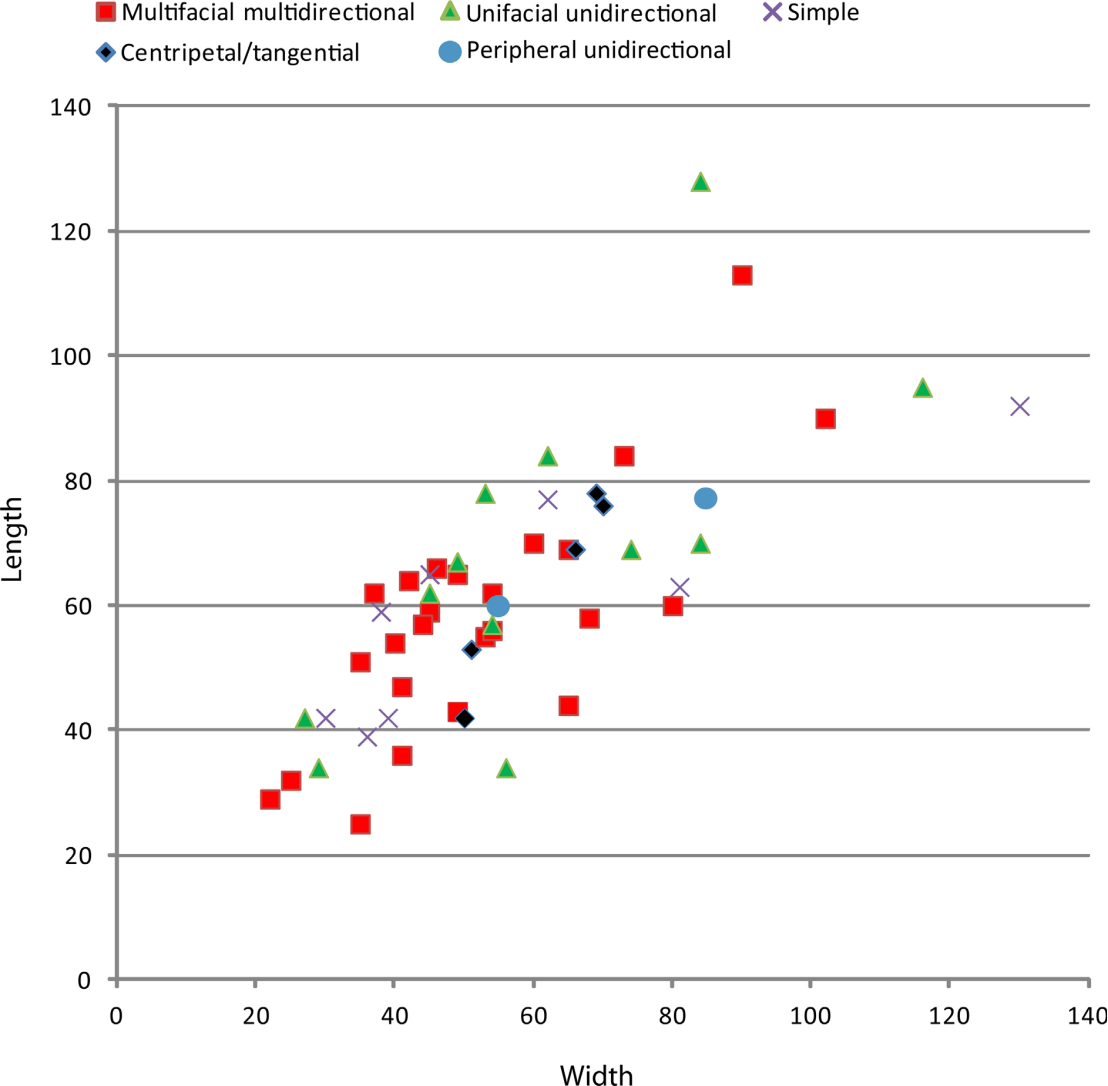
Size distribution (mm) of obsidian cores from Garba IVE-F.

In layers E-F, knapping is devoted exclusively to the production of small- to medium-sized flakes (Figs 6–9; Tables 1 and 2). Except for a few simple cores (n = 8), flaking methods are structured and closely dependent on blank geometry. The most frequent flaking method is irregular multifacial multidirectional exploitation (Fig 6: 5 and Fig 7: 1–4). Cobbles with several flat surfaces were flaked irregularly using any available flaking angle, without preparing a striking platform, to produce the largest feasible number of flakes. Nevertheless, some cores attest to the knapper’s intent to keep to an orthogonal shape while flaking (Fig 7: 3). Besides, a few cores show major flaked surface(s) with unidirectional long flake scars exploited at the beginning of the reduction process. Multifacial multidirectional cores were usually abandoned when their size had been considerably reduced (Fig 10). The flakes have various shapes (Fig 7: 5–11). A few flakes present one or two series of unidirectional flake scars together with multidirectional negative removals, corresponding to multifacial multidirectional cores with one preferred unidirectional flaking surface (Fig 7: 12). The percentage of core edge flakes is high, implying a continuous rotation of the flaking surfaces (Fig 7: 13–15). Unifacial unidirectional exploitation of the longest available surface of elongated cobbles produced flakes whose length exceeded their width (Fig 6: 1 and Fig 8: 3–7, 10). The natural convex surface of cobbles was chosen for detaching flakes by the peripheral unidirectional method over the maximal available extension. The striking platform was a naturally flat surface (Fig 8: 11). Centripetal/tangential exploitation was mostly performed on a flaking surface from a natural peripheral platform, or from a striking platform rectified by only a few removals. Volume and convexity configurations were not managed, recurrence and preparation are absent, there is no evidence of hierarchy. The aim was simply to find a proper technical solution when exploiting (sub)spherical cobbles (Fig 6: 2, 3 and Fig 9: 1, 2, 6). Only one core documents bifacial centripetal exploitation (Fig 6: 2 and Fig 9: 7). The flakes produced by this method are circular, triangular or sub-quadrangular with centripetal or tangential removals on the dorsal face. The butts are natural, plain, dihedral or rarely faceted, wide and thick, and the flaking angle is generally obtuse (Fig 9: 3–5). When the lack of convexities (as on flat cobbles) made it possible to detach only short flakes, the resulting cores are unifacial or bifacial partial ones (Fig 6: 4 and Fig 8: 1, 8).

### Small tool production

**Fig 11:**
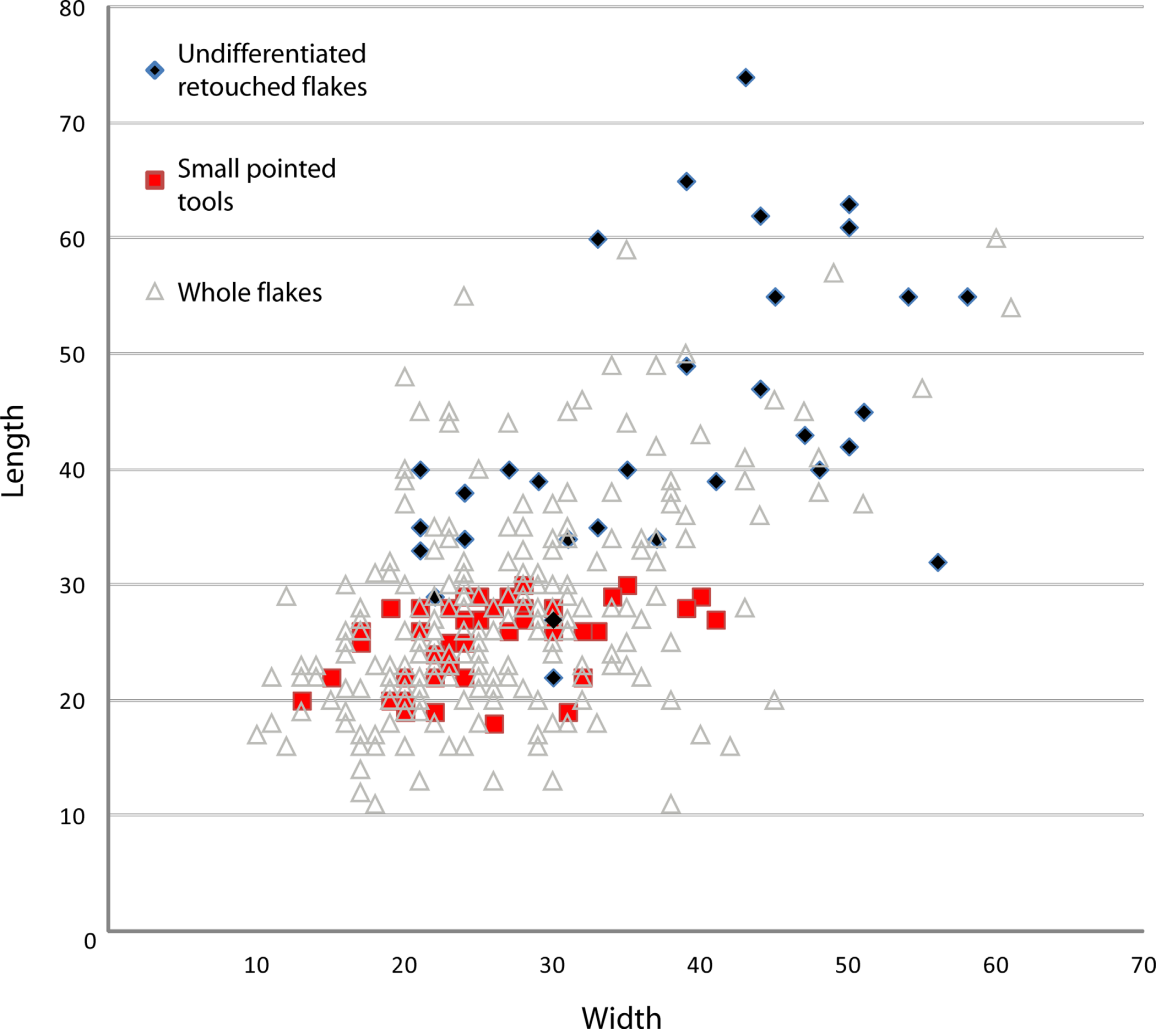
Size distribution (mm) of obsidian whole flakes, small pointed tools, and undifferentiated retouched flakes from Garba IVE-F. Two size groups are visible in the retouched items. These two-dimensional groups belong to two different tool sets, i.e. the undifferentiated retouched tools and the small pointed tools.

**Fig 12:**
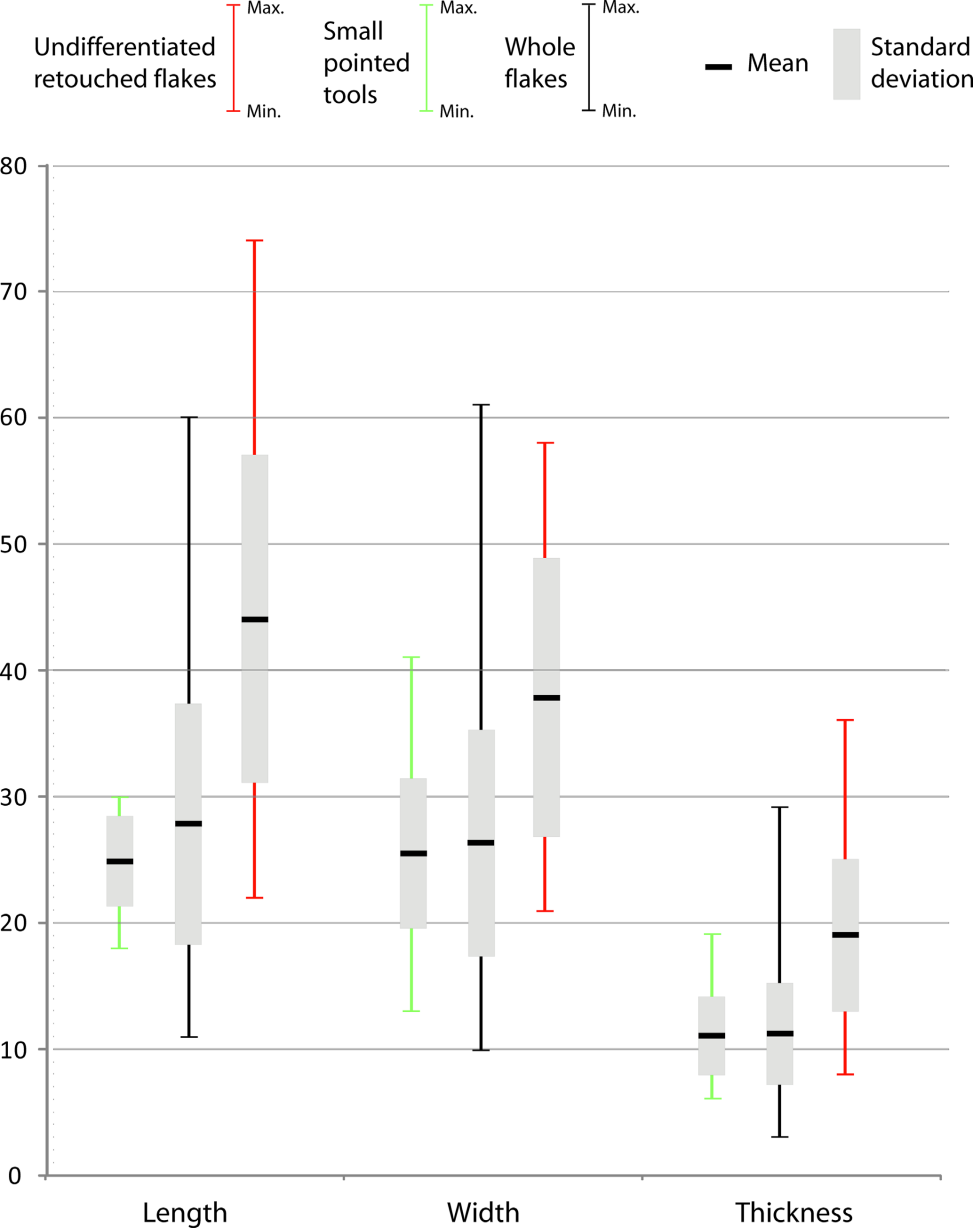
Minimum, maximum, mean, and standard deviation values of small pointed tools, whole flakes, and undifferentiated retouched flakes, grouped by length, width and thickness.

**Fig 13:**
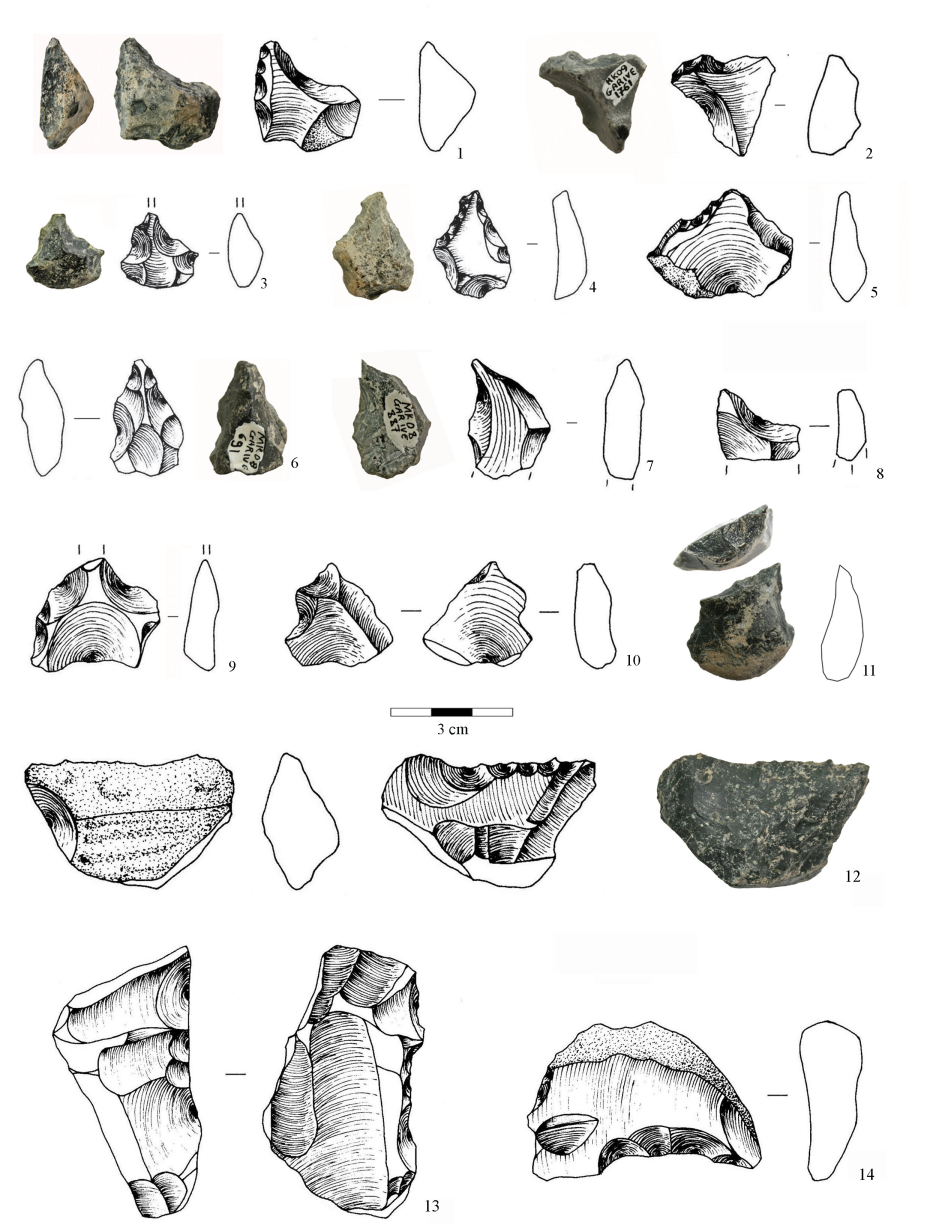
Photographs and drawings of selected pointed obsidian small tools (1–11) and obsidian undifferentiated retouched flakes (12–14) from Garba IVE-F. 1, 11: notch opposite a retouched edge; 2, 3, 6, 9, 10: two or more notches located on two convergent edges; 4: convergent side-scraper; 5: retouched edge opposite a back; 7, 8: notch opposite a back; 12: transversal side-scraper; 13: lateral side-scraper on core edge flake; 14: retouched proximal notch. Drawings by N. Tomei; photos by R. Gallotti.

A relatively large percentage of the whole obsidian flakes had been retouched (31%; n = 73). This development is clearly linked to the use of high-quality raw material, because aside from the obsidian flakes, only one aphyric basalt flake shows marginal retouch. Side-scrapers and notches, as well as small points, were manufactured at Garba IVE-F. They can be grouped in two sets. The first one, identified only in layer E, consists of flakes (n = 32) whose edges were modified by a retouch that did not transform the original blank into any standard form. The retouch is continuous but highly variable, ranging from marginal to invasive. The resulting tools display large dimensional and morphological variability (Figs 11 and 12). There is no close relationship to any specific flaking method. Most blanks are either first flakes, or flakes with natural residual parts on the dorsal face (Fig 13: 12, 14), or core edge flakes (Fig 13: 13).

In contrast, the second set (41 tools) displays a retouch process aimed specifically at producing a small point, modifying the distal part of the blank (Fig 13). The morphological axis of the pointed part either corresponds to the percussion axis or shows a skewed direction.

The pointed shape is produced in different ways: 1) by two or more notches located on two convergent edges (Fig 13: 2, 3, 6, 9, 10); 2) by one or more notches opposite a retouched edge (Fig 13: 1, 11); 3) by one or more notches opposite a (natural) back (Fig 13: 7, 8); 4) by a retouched edge opposite a back (Fig 13: 5); 5) by a convergent side-scraper (Fig 13: 4). Accordingly, we are dealing with some degree of standardization, intended here as “the adoption of generally accepted uniform procedures, dimensions, materials, or parts that directly affect the design of a product” [50]. Following this definition, a standard product is a product that conforms to specifications resulting from the same technical requirements. In the small pointed tools of Garba IVE-F, standardization is expressed by the simultaneous occurrence of: 1) a repetitive intention to shape the distal portion of the flake into a tip; even when the retouch is mostly made by notches, the latter ones are usually multiple and continuous; 2) a repetitive intention to create a convergence, either modifying both edges or modifying one edge and using the technical properties of the other one; and 3) a recurrent search for a small and homogeneous size. While in the other tool set the dimensions range from 22 to 74 mm, in the pointed tools the length is kept tightly between 18 and 30 mm (Fig 11). Besides, in pointed tools the means of length, thickness and width are very close to those of the unmodified whole flakes (Fig 12). The undifferentiated retouched flakes are substantially larger.

Intentional behavior is further proved by: 1) hundreds of small flakes (<1cm) found in the same layers but clearly distinct from natural or knapped fragments of the same size; 2) the lack of any bipolar technique on anvil, which is often responsible for pseudo-retouching [9, 51]; 3) the similar technical marks left by retouch, on both lava and obsidian flakes from later Melka Kunture sites [27]; 4) the lack of any evidence of bioturbation or trampling in the deposit [23]. Summing up, the unusually high number of retouched and often pointed tools at Garba IVE-F is not the outcome of any natural process that altered the edges.

## Discussion

In the case of obsidian as in the case of lavas, small-to-medium flake production is the only technological focus of the assemblages from Garba IV basal sequence (layers E-F) and artifacts are mostly resulting from knapping of obsidian cobbles. Those were abundantly available in Quaternary alluvial deposits, and only few were left unworked in the archaeological layers. East Africa is one of the few areas in the world with an abundance of obsidian outcrops. Nevertheless, this volcanic glass was not available or otherwise never knapped by hominins during the Oldowan and rarely used during the Acheulean. Obsidian was intensely exploited later, starting in the Middle Stone Age, and became dominant in the Late Stone Age [1, 11, 52–54]. Melka Kunture is the only known exception, documenting a continuous and extensive use of obsidian since the very beginning of stone-tool production [26–29, 34].

Cores, which clearly fall into the category of technological waste, never bear any sign of retouch or edge shaping. Cores were generally abandoned either when they were considerably reduced in size, or following the exhaustion of the suitable convexity of the debitage surface(s). The technical patterns belonging to the debitage methods show a systematic adaptation to the geometry of the natural matrixes. The debitage mostly follows the natural angles or rectifies them through few removals in order to obtain angles suitable for knapping. A true preparation of the striking platform is lacking, as well as recurrence, volume/convexities management, hierarchy among surfaces, and modification of the natural blank geometry in order to adopt a specific flaking method. These technical aspects occur for the first time at ~1.5 Ma in the Melka Kunture sequence in the upper layer (D) of Garba IV, together with the emergence of the Large Cutting Tool production [27]. Such innovations within small debitage have been suggested as a proxy for the origin of the Acheulean in East Africa [27, 55–56]. Even if layers E-F at Garba IV are approximately contemporaneous with the oldest early Acheulean sites [57–60], the flaking methods adopted by the knappers were governed by technical structures, skills, control and cognitive abilities similar to those identified at the other Oldowan sites in East Africa [4, 8–10].

Nevertheless, there is also evidence of a specific technical process never recorded before. The high frequency of small tools together with the systematic search for pointed forms are a particular aspect of this industry. Finding standardized retouched pieces in an Oldowan assemblage raises a number of questions. Are they the outcome of cognitive differences between the Oldowan tool-makers at Melka Kunture and the knappers of otherwise similar techno-complexes discovered elsewhere in East Africa? Or are they due to availability of exceptionally high-quality raw material at Melka Kunture? However, aphyric lavas, which are readily available and accessible in local ancient streams, are likewise excellent fine-grained rocks. The flaking methods are the outcome of the same knapping skills displayed in the case of obsidian. Hence, knapping suitability in itself does not explain the observed difference. Were the Oldowan knappers whose output was found in Garba IVE-F unable to produce retouched tools on lava? This is utterly improbable, as aphyric lavas, which were also easily available, are fine-grained rocks with suitable edges for retouch. This is well evidenced in the early Acheulean of Garba IVD some 0.2 Ma later, when retouching modifies both obsidian and lava flakes in the same way [27]. Furthermore, at Garba IVD the retouched pieces can be classified as side-scrapers, denticulates and notches. These tools are unfrequent (5% of the flakes) and do not seem to have been a main objective of the knapping activities. The retouch generally occurs along only one edge, either lateral or transversal, and rarely on two edges. The retouch never modifies the shape of the blank [27].

Thus we assume that the small pointed tools were produced for a specific techno-functional purpose. Unfortunately, the intense hydration undergone by obsidian knapped more ~ 1.7 Ma ago precludes use-wear studies. The purpose of producing standardized small points remains unknown.

Garba IVE-F, at ~1.7 Ma, is the only archaeological evidence ascribed at Melka Kunture to the Oldowan Industrial Complex on the basis of a technological analysis. Gombore IB, dated to > 1.39, and probably penecontemporaneous to Garba IVD [23–24], as well as Karre I, with an uncertain chronostratigraphy, were both related to the Oldowan by Chavaillon [61]. However, this was after a typometrical study only, which had been performed in Eighties of last century. The analysis of Gombore IB and Karre I cannot be compared to the analysis of Garba IVE-F. Accordingly, at Melka Kunture itself we cannot evaluate whereas the small pointed tool production showing a certain degree of standardization is the outcome or not of an Oldowan standardized behavior.

So far, the standardization in the manufacture of small tools described above suggests an occasional technological development probably driven by practical needs, and facilitated by the high knapping suitability of obsidian. Our findings contradict hypotheses whereby increasing numbers of retouched flakes and the emergence of standardization in tool-kits are related to growing technical skills and thus reflect a major step in cultural evolution. These technical/cognitive components are taken worldwide as emerging at the transition from the Oldowan to the Acheulean and also as a hallmark of the Acheulean techno-complexes [62]. We suspect that this is the outcome of a perspective that holds that an otherwise ill-defined Oldowan technology embraces several continents over nearly two million years [63–65]. However, the evidence from Oldowan and early Acheulean sites in East Africa does not support this scenario. As said previously, the introduction of systematic flake modification with standardized aspects documented at ~1.7 Ma at Melka Kunture was not followed by any further production of similar tools in the early Acheulean of Garba IVD [27]. The same occurs at Olduvai middle Bed II [9], even if the overall number of retouched flakes increases through time. There are very few retouched tools at RHS-Mugulud and MHS-Bayasi in Peninj as well as at Gadeb 2E [55–56], where retouch just modifies flake edges. During the later Acheulean at Melka Kunture, small tools continue to be few and standardization is limited to the technical processes of Large Cutting Tool productions [26, 28]. On the Ethiopian highlands and in East Africa in general, standardized small tools start to play the role of cultural markers only in the Middle Stone Age, when both flaking methods and retouching processes came to be involved in the production of specific types of highly performing tools.

## Conclusion

The small tools in Garba IVE-F support the contention that the Oldowan knappers, when driven by functional needs and supported by a highly suitable raw material, were capable of some degree of innovation. The implemented technical solutions, only rarely seen in the archaeological record, appear to have been regularly used or standardized at Garba IVE-F. This capacity was rooted in a good knowledge of the suitability of raw materials. The small tool production at ~1.7 Ma, at a time when the Acheulean was already emerging elsewhere in East Africa [57–60], adds new information on the technical skills and flexibility developed by the Oldowan knappers. The many small pointed tools, unknown elsewhere, also add to the growing amount of evidence of Oldowan techno-economic variability, further challenging the view that early stone knapping was static over hundreds of thousands of years.

However, why small-tool production did not become part of the emerging Acheulean remains an open question.
